# All-optical image denoising using a diffractive visual processor

**DOI:** 10.1038/s41377-024-01385-6

**Published:** 2024-02-04

**Authors:** Çağatay Işıl, Tianyi Gan, Fazil Onuralp Ardic, Koray Mentesoglu, Jagrit Digani, Huseyin Karaca, Hanlong Chen, Jingxi Li, Deniz Mengu, Mona Jarrahi, Kaan Akşit, Aydogan Ozcan

**Affiliations:** 1 Electrical and Computer Engineering Department, University of California, Los Angeles, CA 90095 USA; 2 Bioengineering Department, University of California, Los Angeles, CA 90095 USA; 3 California NanoSystems Institute (CNSI), University of California, Los Angeles, CA 90095 USA; 4 University College London, Department of Computer Science, London, United Kingdom

**Keywords:** Optical physics, Imaging and sensing, Applied optics, Transformation optics

## Abstract

Image denoising, one of the essential inverse problems, targets to remove noise/artifacts from input images. In general, digital image denoising algorithms, executed on computers, present latency due to several iterations implemented in, e.g., graphics processing units (GPUs). While deep learning-enabled methods can operate non-iteratively, they also introduce latency and impose a significant computational burden, leading to increased power consumption. Here, we introduce an analog diffractive image denoiser to all-optically and non-iteratively clean various forms of noise and artifacts from input images – implemented at the speed of light propagation within a thin diffractive visual processor that axially spans <250 × λ, where λ is the wavelength of light. This all-optical image denoiser comprises passive transmissive layers optimized using deep learning to physically scatter the optical modes that represent various noise features, causing them to miss the output image Field-of-View (FoV) while retaining the object features of interest. Our results show that these diffractive denoisers can efficiently remove salt and pepper noise and image rendering-related spatial artifacts from input phase or intensity images while achieving an output power efficiency of ~30–40%. We experimentally demonstrated the effectiveness of this analog denoiser architecture using a 3D-printed diffractive visual processor operating at the terahertz spectrum. Owing to their speed, power-efficiency, and minimal computational overhead, all-optical diffractive denoisers can be transformative for various image display and projection systems, including, e.g., holographic displays.

## Introduction

Image denoising is a fundamental problem encountered in various fields, such as computational imaging and displays^1^, computer vision^2^, and computer graphics^3–5^. For example, in computational imaging, noise removal from images is used to mitigate the effects of various sources of noise and artifacts, e.g., image sensors, channel transmission, and environmental conditions^6,7^. Similarly, within the realm of computer graphics, image denoising is crucial for reducing the low-sampling related image rendering artifacts frequently encountered in real-time graphics processing applications^8–10^.

Over the past several decades, numerous algorithms have been developed for image noise removal^11–13^. Apart from his renowned contributions to the birth of holography, Dennis Gabor proposed one of the earliest methods for image denoising, involving the Gaussian smoothing of noisy images^14^. A plethora of other algorithms emerged for image denoising, including, e.g., Wiener filtering^2^, anisotropic filtering^15^, total variation (TV) denoising^16^, denoising by soft-thresholding^17^, bilateral filtering^18^, non-local means denoising^19^, block-matching and 3D filtering (BM3D)^20^, and among many others^11–13^. While quite powerful, these classical denoising techniques often need many iterations for their inference, making them less suitable for real-time applications. Deep Neural Networks (DNNs) have also significantly impacted the field of image denoising, especially in the last decade^21,22^. These artificial DNNs have many parameters that are stochastically optimized (trained) using supervised learning with a large number of noisy-clean image pairs forming the training image set. After their training, DNNs generally operate in a non-iterative feed-forward fashion and have shown remarkable performance advantages for image denoising of unknown input images, never seen before^23–33^. It was also reported that DNN-based image denoisers could be used for real-time applications, including interactive Monte Carlo rendering^8,9,34–36^. Despite the recent improvements in modern graphics processing units (GPUs), achieving interactive speeds in Monte Carlo rendering necessitates working with low spatial sampling, resulting in artifacts in the rendered images. DNN-based denoisers have been proposed to mitigate such artifacts for real-time applications, demanding the use of relatively costly and resource-intensive GPUs^37,38^.

**Fig. 1:**
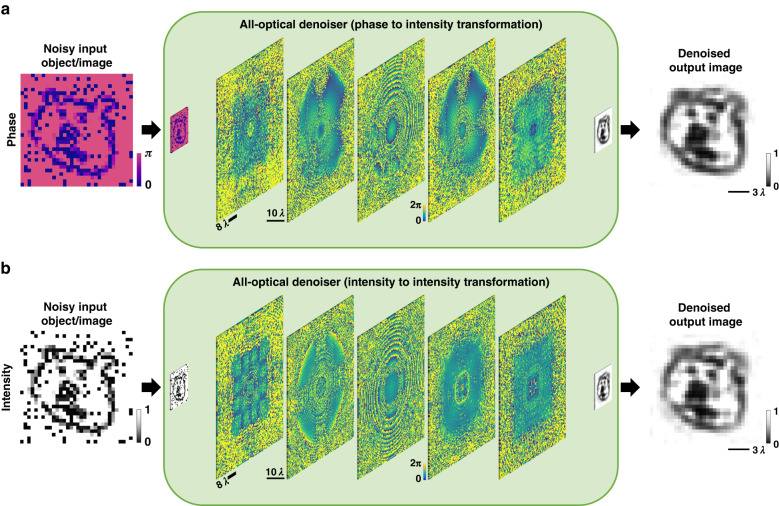
**Schematic of all-optical diffractive image denoiser networks**. **a** 5-layer diffractive denoiser operating on noisy input phase images/objects. **b** 5-layer diffractive denoiser operating on noisy input intensity images/objects

Here, we report an analog diffractive image denoiser (Fig. 1) designed to all-optically process noisy phase or intensity images to filter out noise at the speed of light propagation through a thin diffractive visual processor – optimized using deep learning. Our diffractive denoiser framework consists of successive passive modulation layers that are each transmissive; this diffractive architecture forms a coherent image processor that all-optically scatters out the optical modes representing various forms of noise and spatial artifacts at the input images, causing them to miss the output image Field-of-View (FoV), while passing the optical modes representing the desired spatial features of the input objects with minimal loss and aberrations, forming denoised images at the output FoV without any digital computation in its blind inference. Following its one-time supervised learning-based training performed on a computer, a diffractive image denoiser can work at any part of the electromagnetic spectrum by scaling the dimensions of its optimized diffractive features in proportion to the wavelength of light (λ), eliminating the need to redesign its layers for different wavelengths of operation.

We demonstrate the capabilities of this analog diffractive image denoiser framework on both phase and intensity images, mitigating salt and pepper noise and low-sampling related spatial image artifacts. Our analyses show that these all-optical denoisers successfully filter out various types of image noise or artifacts at the input using a thin diffractive processor^39–44^ that axially spans <250 × λ, while achieving a decent output power efficiency of ~30–40%. For a proof-of-concept, the presented diffractive denoiser framework was also experimentally validated at the terahertz spectrum for removing salt-only random noise in intensity input images using 3D-printed diffractive layers optimized via deep learning. This physical image denoiser framework presents a rapid and power-efficient solution for all-optical filtering of image noise or artifacts, and can potentially be used for holographic displays and projectors operating at different parts of the electromagnetic spectrum.

## Results

**Fig. 2:**
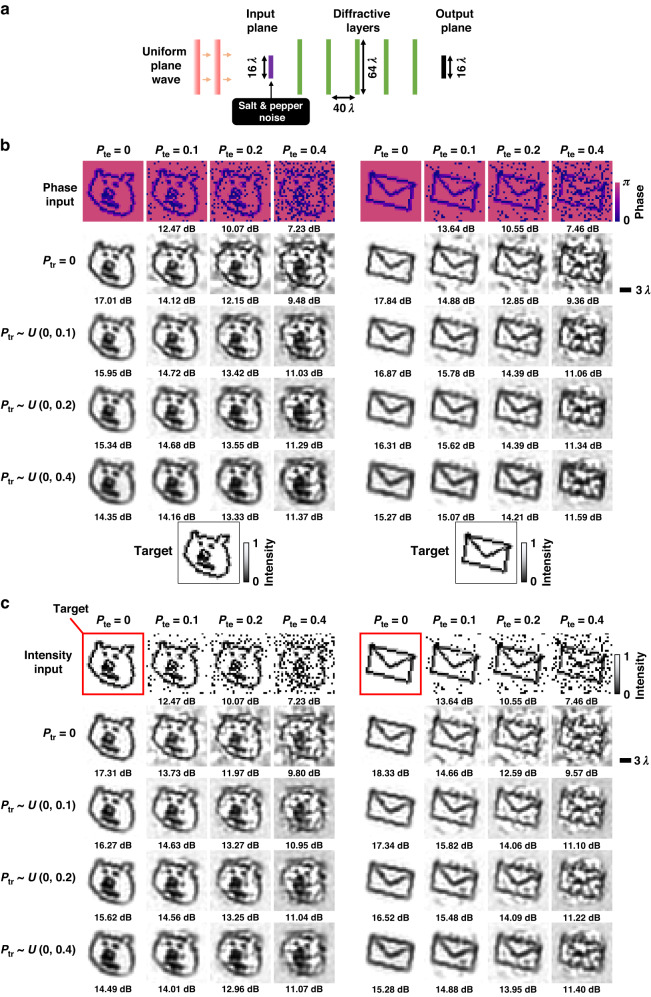
**Simulation results of 5-layer all-optical diffractive image denoisers for filtering out salt & pepper noise. a** Optical layout of the diffractive image denoisers operating on phase or intensity input images. **b** All-optical image denoising results of different diffractive image denoisers with phase-encoded inputs, which are trained using ***P***_***tr***_ drawn uniformly from different intervals. The PSNR value for each case is shown beneath the corresponding output image. **c** All-optical image denoising results of different diffractive denoisers with intensity-encoded inputs, trained using ***P***_***tr***_ drawn uniformly from different intervals

In this manuscript, the terms “diffractive visual processor”, “diffractive image denoiser”, “diffractive optical network”, and “all-optical image denoiser” are interchangeably used. Figure 1 illustrates the schematic of two different diffractive image denoisers trained to all-optically filter out salt and pepper noise from noisy phase or intensity input images; the first one of these diffractive image denoisers (Fig. 1a) is trained to perform phase-to-intensity transformations, whereas the second one (Fig. 1b) is trained to perform intensity-to-intensity transformations between the input and output FoVs. A comprehensive analysis of the all-optical image denoising performances of these trained diffractive denoiser designs under various levels of salt and pepper noise is demonstrated in Fig. 2. In these numerical analyses, each one of the all-optical image denoisers has 5 diffractive layers, which were optimized/trained using the *tiny quickdraw* dataset^45^. The input illumination is considered to be a uniform plane wave (monochromatic and spatially coherent), and the noisy input images to be filtered are in the form of either phase-encoded or intensity-encoded images (see Fig. 2a). For each input encoding type (phase/intensity), different diffractive denoisers were trained using noise probabilities (***P***_***tr***_) sampled uniformly from $U(0,\rho )$ where $\rho \in \left\{0.1,0.2,0.4\right\}$; ***P***_***tr***_ determines the ratio of the image pixels affected by noise relative to the overall pixel count of the image; see the Methods for details. These training noise probabilities (***P***_***tr***_) were randomly sampled for each batch of the input images during each epoch of the training, and the noise-free case (${{\boldsymbol{P}}}_{{\boldsymbol{tr}}}=0$) in Fig. 2b, c corresponds to our baseline designs trained with input images free from noise or artifacts. All the trained models were blindly tested using the tiny *quickdraw* test dataset for different test noise probabilities (***P***_***te***_). Peak-Signal-to-Noise Ratio (PSNR) and Structural Similarity Index Measure (SSIM) were used as image quality metrics to quantify the all-optical denoising performance of the trained models^46^. Further information regarding the architecture of the diffractive image denoisers, the noise models, the training loss functions, the datasets, and other aspects of our implementation are reported in the Methods section.

Figure 2b illustrates the all-optical image denoising results of diffractive denoisers trained for noisy phase-encoded input images (salt and pepper noise). The output intensities shown for two test images illustrate the success of the trained diffractive image denoisers (with ${{\boldsymbol{P}}}_{{\boldsymbol{tr}}}\,{\boldsymbol{ \sim }}\,U(0,\rho )$ where $\rho {{\in }}\left\{\mathrm{0.1,0.2,0.4}\right\}$) compared to a conventional diffractive imager trained without noise (i.e., ${{\boldsymbol{P}}}_{{\boldsymbol{tr}}}\,{\boldsymbol{=}}\,0$). For instance, the denoising performance of the diffractive model trained using ${{\boldsymbol{P}}}_{{\boldsymbol{tr}}}\,{\boldsymbol{ \sim }}\,U(\mathrm{0,0.2})$ demonstrates superior performance for phase-encoded test images created with ${{\boldsymbol{P}}}_{{\boldsymbol{te}}}\,{\boldsymbol{=}}\,{0.1,0.2},\,{\rm{and}}\,0.4$, achieving average PSNR improvements of 0.65, 1.47, and 1.90 dB, respectively, when compared to the diffractive imager trained without noise, ${{\boldsymbol{P}}}_{{\boldsymbol{tr}}}\,{\boldsymbol{=}}\,0$. A similar conclusion can be drawn for all-optical filtering of the intensity-encoded noisy images reported in Fig. 2c. The diffractive image denoiser trained using ${{\boldsymbol{P}}}_{{\boldsymbol{tr}}}\,{\boldsymbol{ \sim }}\,U\left({0,0.2}\right)$ exhibits an improved denoising performance when compared to the baseline diffractive imager (${{\boldsymbol{P}}}_{{\boldsymbol{tr}}}\,{\boldsymbol{=}}\,0$), achieving average output image PSNR improvements of 0.83, 1.39, and 1.45 dB for different noise levels of ***P***_***te***_ **=** 0.1, 0.2, and 0.4, respectively.

We also compared our diffractive image denoisers against a 4-f lens-based spatial filtering system, one of the common ways of denoising in optical setups, as detailed in Supplementary Fig. S1. In this comparison, diffractive denoisers operating on noisy intensity images shared the same input numerical aperture (NA) with the 4-f filter system, as depicted in Supplementary Fig. S1a, b. For low-pass filtering, the 4-f system employed a transparent circular aperture with a diameter of ${\boldsymbol{\delta }}\times{64}{\lambda}$ where ***δ*** represents the level of spatial filtering. As shown in Supplementary Fig. S1c, our diffractive processors outperform the 4-f system in denoising intensity images that are impacted by salt and pepper noise. For example, the diffractive image denoiser trained with ${{\boldsymbol{P}}}_{{\boldsymbol{tr}}}\,{\boldsymbol{ \sim }}\,U\left(\mathrm{0,0.2}\right)$ demonstrated superior denoising performance compared to the 4-f system with ${\boldsymbol{\delta }}\,{\boldsymbol{=}}\,{0.6}$, achieving average output image PSNR improvements of 2.76, 2.52, 2.15, and 1.49 dB for different test noise levels of ${{\boldsymbol{P}}}_{{\boldsymbol{te}}}{\boldsymbol{=}}\mathrm{0,0.1,0.2},{\rm{and}}\,0.4$, respectively. This competitive performance of diffractive denoisers stems from their capability to execute any arbitrary complex-valued linear transformation between the input and output FoVs, covering spatially varying point spread functions. In fact, this feature represents a superset compared to all types of spatially invariant imaging systems, including 4-f filtering systems.

These numerical results reported in Fig. 2 and Supplementary Fig. S1 demonstrate the versatility of the all-optical image denoiser framework to filter out salt and pepper noise present at the input phase or intensity images. These diffractive image denoisers effectively learn to filter out the spatial modes that statistically represent the targeted noise features, while successfully transferring the spatial modes representing the desired features of the input objects, forming denoised intensity images at the output FoV with minimal optical loss and aberrations. In this sense, a diffractive image denoiser can be considered a 3D spatial filter composed of successive phase gratings structurally optimized through supervised deep learning to physically couple out undesired spatial modes of targeted noise features, causing them to miss the output image FoV.

**Fig. 3:**
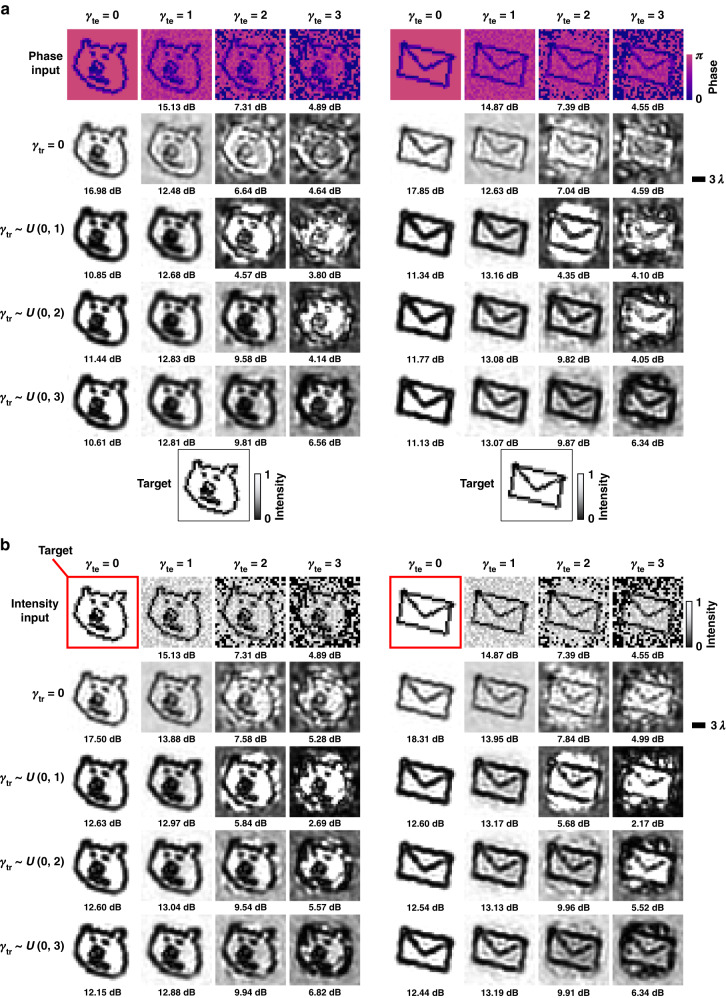
**Simulation results of 5-layer all-optical diffractive image denoisers for filtering out Monte Carlo low-sampling artifacts. a** All-optical image denoising results of different diffractive denoisers with phase-encoded inputs, which are trained using ***γ***_***tr***_ sampled uniformly from different intervals. The PSNR value for each case is shown beneath the respective output image. **b** All-optical image denoising results of different diffractive denoisers using intensity-encoded inputs, which are trained using ***P***_***tr***_ drawn uniformly from different intervals

In addition to salt and pepper noise, we also designed diffractive image denoisers to mitigate image artifacts stemming from the Monte Carlo-based low-sampling image renderings, as depicted in Fig. 3. In this analysis, we report the results of different diffractive denoisers, which were trained using noise rates (***γ***_***tr***_) sampled uniformly from $U(0,\rho )$ where $\rho \in\left\{1,2,3\right\}$ for both phase-encoded and intensity-encoded input images. ***γ***_***tr***_ indicates the noise rate of the Monte Carlo image renderings, as detailed in the Methods section. Diffractive models with ***γ***_***tr***_ = 0 define our baseline, trained with noise-free input images. The denoising capabilities of these diffractive models were blindly tested for various levels of test noise, ${{\boldsymbol{\gamma }}}_{{\boldsymbol{te}}}\in \left\{0,1,2,3\right\}$; see the Methods section for details. The results of these analyses are reported in Fig. 3a, b, which demonstrate the advantages of all-optical image denoisers for both phase-encoded and intensity-encoded input images, further supporting the conclusions of the earlier analyses in Fig. 2. For example, Fig. 3a present the results of the diffractive models trained using phase-encoded images with ${{\boldsymbol{\gamma }}}_{{\boldsymbol{tr}}}\,{\boldsymbol{ \sim }}\,U({0,3})$, which outperform the diffractive imager trained with ${{\boldsymbol{\gamma }}}_{{\boldsymbol{tr}}}=0$ for various test images, improving the average PSNR values by 0.39, 3, and 1.84 dB for ${{\boldsymbol{\gamma }}}_{{\boldsymbol{te}}}\,{\boldsymbol{=}}\,{1,2},{\mathrm{and}}\,3$, respectively. Similarly, Fig. 3b demonstrates the success of the all-optical diffractive denoiser models trained using intensity-encoded input images, achieving average PSNR improvements of, e.g., 2.22 and 1.45 dB for ${{\boldsymbol{\gamma }}}_{{\boldsymbol{te}}}{\boldsymbol\,\,{=}}\,\,2\,\mathrm{and}\,3$, respectively, in comparison to the diffractive imager trained using ${{\boldsymbol{\gamma }}}_{{\boldsymbol{tr}}}=0$.

**Fig. 4:**
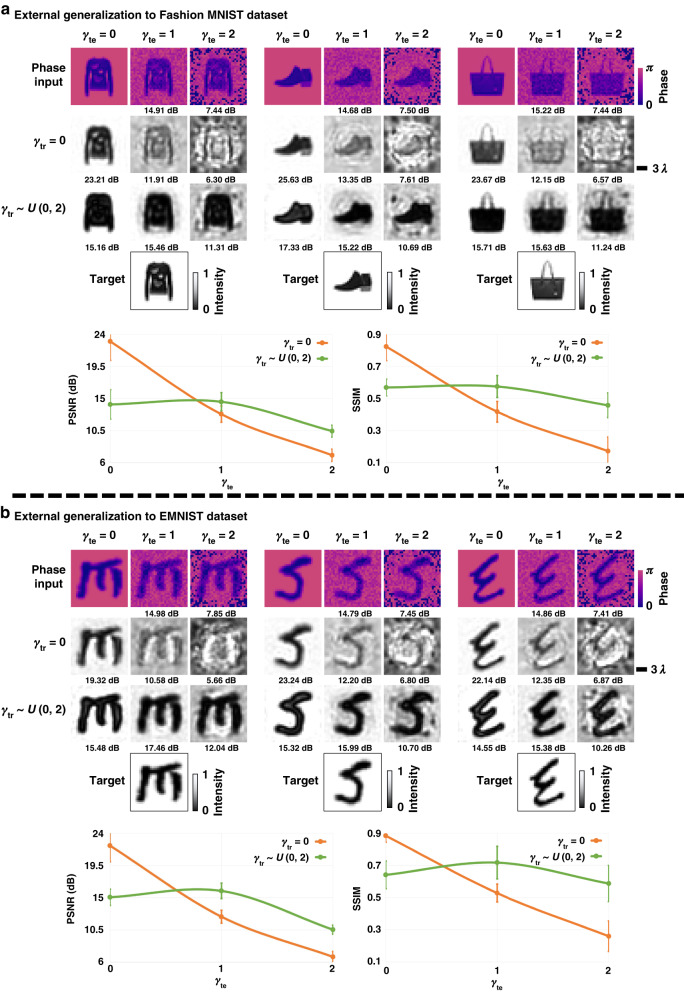
**External generalization performance of 5-layer all-optical diffractive image denoisers for Monte Carlo low-sampling artifact removal**. The diffractive image denoiser using phase-encoded inputs is trained with the *tiny quickdraw* dataset. **a** All-optical image denoising results on three images randomly selected from the Fashion MNIST test dataset and the average PSNR and SSIM values on the same test dataset as a function of ***γ***_***te***_. The PSNR value for each case is shown beneath the respective output image. **b** All-optical image denoising results on three images of the EMNIST test dataset and the average PSNR and SSIM values on the same dataset as a function of ***γ***_***te***_

These results reported in Figs. 2 and 3 demonstrate the *internal* generalization capabilities of the trained diffractive denoisers as the test images (although never seen before) were acquired from the same dataset (*tiny quickdraw*). To explore the *external* generalization of the all-optical denoisers to different datasets containing images with distinct spatial features, we conducted additional tests using Fashion MNIST and EMNIST image datasets^47,48^, as illustrated in Fig. 4. The trained diffractive image denoiser, which mitigates the low-sampling artifacts of Monte Carlo renderings (${{\boldsymbol{\gamma }}}_{{\boldsymbol{tr}}}\,{\boldsymbol{ \sim }}\,U({0,2})$) from phase-encoded input images, and the baseline diffractive imager trained with ${{\boldsymbol{\gamma }}}_{{\boldsymbol{tr}}}=0$ are both tested with noisy input images with different levels of noise (${{\boldsymbol{\gamma }}}_{{\boldsymbol{te}}}{{\in }}\left\{{0,1,2}\right\}$). The average PSNR and SSIM values calculated across the corresponding test datasets confirm the external generalization capabilities of the all-optical image denoisers. For example, as illustrated in Fig. 4a, the diffractive image denoiser trained with ${{\boldsymbol{\gamma }}}_{{\boldsymbol{tr}}}\,{\boldsymbol{ \sim }}\,U({0,2})$ achieves average PSNR (SSIM) improvements of 1.74 dB (0.157) for ${{\boldsymbol{\gamma }}}_{{\boldsymbol{te}}}\,{\boldsymbol{=}}\,1$ and 3.37 dB (0.285) for ${{\boldsymbol{\gamma }}}_{{\boldsymbol{te}}}\,{\boldsymbol{=}}\,2$, when compared to the baseline diffractive imager trained without noise (${{\boldsymbol{\gamma }}}_{{\boldsymbol{tr}}}=0$). A similar analysis is reported in Supplementary Fig. S2, which further confirms the external generalization capabilities of diffractive image denoisers trained with salt and pepper noise. In addition to this external generalization performance, the test images from the Fashion MNIST dataset also highlight the success of diffractive denoisers in processing gray-scale images never seen before. In fact, further improvements in the denoising performance for these gray-scale Fashion MNIST images are attainable by training a diffractive image denoiser from scratch using the Fashion MNIST training dataset (see Supplementary Fig. S3a). For example, the diffractive denoiser trained using the Fashion MNIST dataset (${{\boldsymbol{P}}}_{{\boldsymbol{tr}}}\,{\boldsymbol{ \sim }}\,U\left({0,0.2}\right)$) achieves an average PSNR improvement of 1.39 dB compared to the diffractive denoiser trained using the *tiny quickdraw* dataset (${{\boldsymbol{P}}}_{{\boldsymbol{tr}}}\,{\boldsymbol{ \sim }}\,U\left({0,0.2}\right)$) when both models are tested using images of the Fashion MNIST test dataset at a noise level of ${{\boldsymbol{P}}}_{{\boldsymbol{te}}}\,{\boldsymbol{=}}\,0.1$. In addition to these analyses, Supplementary Fig. S3b demonstrates the versatility of the diffractive image denoiser framework by successfully mitigating other types of noise, such as a modified version of the salt and pepper noise with 4 noise levels (see the Methods section for details).

**Fig. 5:**
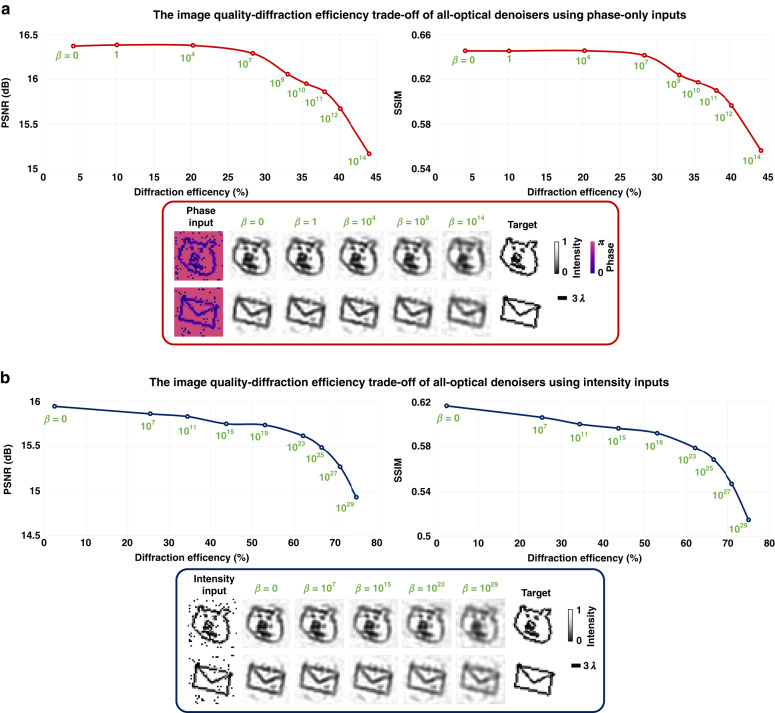
**Quantitative performance of 5-layer diffractive image denoisers as a function of the output diffraction efficiency for phase-encoded and intensity-encoded inputs**. The weight of the diffraction efficiency loss term (*β*) is varied to train diffractive image denoisers with different output efficiencies. These all-optical image denoisers for each input type are trained using the *tiny quickdraw* training dataset under the salt and pepper noise with ***P***_***tr***_ sampled uniformly from the interval *U*(0,0.2). Subsequently, the trained models are tested on the *tiny quickdraw* test dataset, affected by the salt and pepper noise with ***P***_***te***_ = 0.1. **a** All-optical image denoising performance of the diffractive denoisers with phase-encoded inputs as a function of the average output diffraction efficiency. **b** All-optical image denoising performance of the diffractive denoisers with intensity-encoded inputs as a function of the average output diffraction efficiency

One of the essential characteristics of all-optical image denoisers, as well as other diffractive visual processors, is their output diffraction efficiency. In the previously demonstrated results reported so far, the all-optical image denoising performance of these diffractive models was achieved without employing a training loss term to penalize low diffraction efficiency at the output FoV. To understand the trade-off between the output diffraction efficiency and the image quality, we conducted additional analysis reported in Fig. 5. As detailed in the Methods section, we adjusted the output diffraction efficiency of an image denoiser by varying the weight (*β*) of the diffraction efficiency loss term. During the training process of each diffractive image denoiser model, noisy input images (*tiny quickdraw* dataset) were used, after being subjected to salt and pepper noise with a noise probability (***P***_***tr***_) sampled uniformly using $U(\mathrm{0,0.2})$. These image denoiser models trained with various *β* values were subsequently tested on images that were affected by salt and pepper noise with a noise probability of ${{\boldsymbol{P}}}_{{\boldsymbol{te}}}\,{\boldsymbol{=}}\,0.1$. As depicted in Fig. 5a, for the denoising of phase-encoded images, an all-optical diffractive denoiser can achieve ~28% diffraction efficiency with negligible degradation in its output image quality (~0.08 dB and ~0.004 decrease in the average PSNR and SSIM values, respectively). Similarly, for intensity-encoded images, all-optical denoisers can be designed to have up to ~34% diffraction efficiency while incurring a negligible decrease in output image quality (e.g., ~0.11 dB and ~0.016 in the average PSNR and SSIM values, respectively); see Fig. 5b. While a 4-f low-pass filtering system can in general achieve a much better diffraction efficiency, it is important to note that the presented diffractive framework outperforms these 4-f systems in denoising performance as illustrated in Supplementary Fig. S1. Moreover, 4-f systems are considerably bigger when compared to the thickness of the presented diffractive denoisers that axially span <250 × λ.

**Fig. 6:**
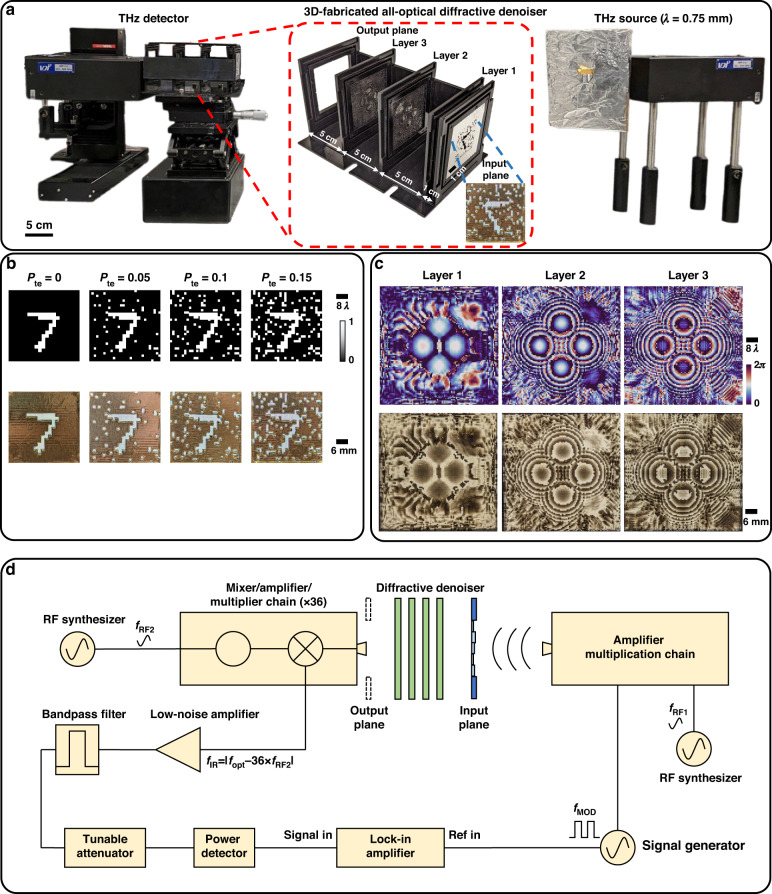
**Experimental setup for a 3-layer diffractive image denoiser. a** Photograph of the experimental setup including the 3D-fabricated all-optical image denoiser trained for noisy intensity images. **b** Intensity profiles of an image example impacted by various levels of salt-only noise (***P***_***te***_) and their photographs after their 3D-fabrication. **c** Phase profiles of the trained diffractive image denoiser layers and their photographs after 3D-fabrication. **d** Schematic of the experimental setup using continuous wave THz illumination (λ = ~0.75 mm)

For an experimental proof-of-concept of the presented technique, we built a 3-layer diffractive image denoiser that was trained for noisy intensity images with salt-only noise, with the noise probability (***P***_***tr***_) uniformly sampled from the interval $U(\mathrm{0,0.2})$. As depicted in Fig. 6a, the resulting diffractive design was then fabricated and precisely aligned for experimental testing using a single-pixel THz detector and a continuous-wave THz illumination source (λ = ~0.75 mm). Figure 6b shows sample binary intensity images of a handwritten letter under different levels of salt-only noise, along with their photographs after the fabrication. Furthermore, the phase profiles of the trained layers of the diffractive image denoiser and photographs of these layers after their fabrication through 3D printing are illustrated in Fig. 6c. During the training of this diffractive model, small random 3D misalignments were introduced to the positions of the diffractive layers to ensure a physically resilient design, which is also referred to as the “vaccination” of the diffractive model^49,50^. Also, an additional loss term for the output diffraction efficiency was incorporated in the design of the diffractive denoiser, resulting in an efficiency of ~2%. The trained diffractive denoiser was also compared against a standard diffractive imager using the same physical configuration and optimization settings, trained with noise-free images; Supplementary Fig. S4 demonstrates the improved denoising performance of our experimental diffractive design trained with noisy images compared with this noise-free design.

**Fig. 7:**
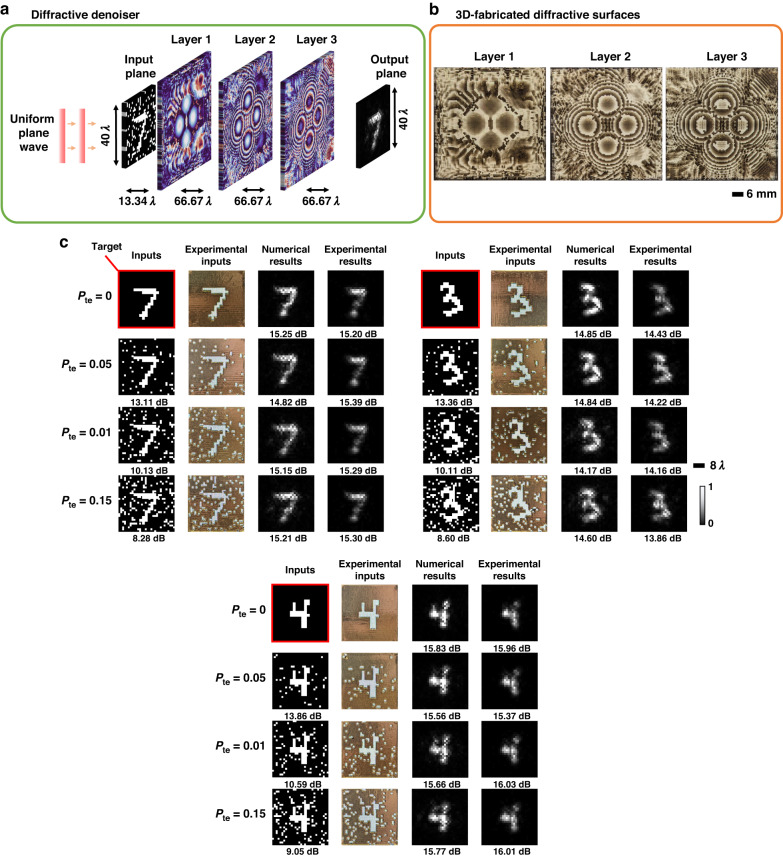
**Experimental results of the all-optical diffractive image denoiser. a** Layout of the diffractive image denoiser with 3 transmissive layers. **b** Photographs of 3D-fabricated layers of the trained diffractive image denoiser. **c** Experimental and numerical image denoising performance of the designed diffractive denoiser under different levels of salt-only noise (***P***_***te***_). The PSNR value for each case is shown beneath the respective image. Experimental results were normalized before the PSNR calculation

The schematic of our experimental setup is depicted in Fig. 6d; see the Methods section for further details. The trained diffractive image denoiser model was experimentally tested on several binary intensity images with various levels of salt-only noise, determined by different noise probabilities (${{\boldsymbol{P}}}_{{\boldsymbol{te}}}{{\in }}\left\{{0,0.05,0.1,0.15}\right\}$). Figure 7 provides the optical layout and the experimental results of the 3-layer diffractive denoiser using these noisy inputs. We observe a very good concordance between the numerical and experimental results presented in Fig. 7c, validating the accuracy and resilience of the 3D-fabricated all-optical diffractive image denoiser. The success of these measurements provides an experimental proof-of-concept of the presented framework for all-optical image denoising. Moreover, the denoising performance of diffractive processors on more extreme, unrecognizable images is depicted in Supplementary Fig. S3a. For example, at a noise level of ${{\boldsymbol{P}}}_{{\boldsymbol{te}}}\,{\boldsymbol{=}}\,0.4$, the “T-shirt” phase image from the Fashion MNIST test dataset becomes almost unrecognizable for human perception, and the diffractive denoiser trained using ${{\boldsymbol{P}}}_{{\boldsymbol{tr}}}\,{\boldsymbol{ \sim }}\,U({0,0.4})$ successfully recovers most parts of the image, mitigating the input noise.

While the results depicted in Fig. 7c showcase the experimental proof-of-concept of the all-optical image denoising framework, the experimental performance is relatively inferior compared to the numerical results presented earlier. This discrepancy is primarily due to the reduced degrees of freedom inherent in our experimental design. To create a misalignment-resilient system with sufficient output diffraction efficiency, a vaccination strategy with an additional loss term was used during the training of the experimental model (as detailed in the Methods section). These measures, however, led to a reduction in the effectively available degrees of freedom of the diffractive denoiser model. Additionally, fabrication-related imperfections in the physical system also impact the image-denoising performance of the diffractive model. By further improving the 3D fabrication resolution and alignment precision, the performance of these physical diffractive denoisers can be significantly enhanced.

## Discussion

We introduced a deep learning-enabled diffractive image denoiser framework capable of addressing various forms of noise inherent to different input types, e.g., phase or intensity images. In our analyses, the all-optical denoisers were used to remove both salt and pepper noise and the spatial artifacts originating from the Monte Carlo low-sample image renderings that are typically addressed using nonlinear filters and deep neural networks running on GPUs. The presented diffractive image denoisers successfully filter out these different types of noise at the input using analog processing of the input object waves; this process effectively couples out the characteristic modes that statistically represent the noise features using the sub-wavelength phase structures of the diffractive layers optimized through deep learning. These phase structures are also optimized to cause minimal optical loss and aberrations for the traveling waves that represent the characteristic modes of the input objects. In this sense, the diffractive image denoiser can be considered a smart analog spatial mode filter composed of successive phase gratings with a lateral pitch of ~λ/2. Additionally, these all-optical image denoisers do not consume any power during the filtering operation except for the illumination light that diffracts through passive layers. Regarding the output diffraction efficiency, our findings reveal that these analog image denoisers can achieve e.g., ~30–40% power efficiency without significantly compromising their image denoising performance. The presented diffractive image denoising framework was also demonstrated experimentally, using a 3D-printed diffractive model, successfully removing salt-only noise from input images as illustrated in Figs. 6 and 7.

The principles underlying diffractive image denoisers can be adapted to suppress other types of noise, including speckle, which is a common issue in holography and coherent imaging/sensing in general. Key factors causing speckle noise include high temporal and spatial coherence of illumination sources, along with the uneven phase profiles found in computer-generated holography methods^51,52^. While reducing the coherence of the light source can mitigate speckle noise, this is less desirable due to its adverse effects on the sharpness and resolution of the reconstructed holographic images^52^. Deep learning-optimized diffractive processors show promise in direct suppression of speckle noise under coherent illumination conditions^51^. In principle, diffractive processors can also be designed to function under partially coherent or incoherent illumination to mitigate different forms of noise with different statistical features^53,54^.

The fundamental components of the presented image denoisers are diffractive layers, consisting of trainable phase structures with a lateral feature size of ~λ/2. The physical communication and collaboration among these sub-wavelength phase structures across different layers are crucial in the functionality of the diffractive image denoising process. For example, Fig. 1 provides a visual representation of various diffractive layers from different image denoisers, each working on noisy phase or intensity input images. While the overall characteristics and topology of these diffractive layers may appear similar, it is the diversity and the full utilization of all the degrees of freedom in these sub-wavelength phase features that collectively achieve the desired image-denoising task.

In our analyses and results, the denoising capabilities of the diffractive denoisers have been demonstrated for the salt and pepper noise and the low-sampling artifacts of Monte Carlo image renderings. Especially for real-time imaging applications, the removal of noisy Monte Carlo renderings is a critical challenge, which has led to the development of various deep learning-based digital image denoisers^8,9,34–36^. Compared to these digital approaches, the all-optical analog operation of diffractive image denoisers enables the processing of input images as the light diffracts through very thin optical elements that axially span <250× λ; this ultra-high speed and power efficiency of diffractive image denoisers would especially be important for real-time image processing applications. For comparison, deep learning-enabled vision networks, as demonstrated in refs. ^55,56^, typically demonstrate a latency of ~60 ms^57^. Our diffractive image processors can process each image in sub ns, consuming minimal power except for the illumination light and operating without the need for digital storage. Although denoisers based on electronic neural networks achieve state-of-the-art denoising performance, the rapid processing and low-power consumption of diffractive image denoisers offer notable advantages, especially for their stand-alone utilization for ultra-fast processing and hybrid vision systems that combine diffractive processors and electronic neural networks^37,58–60^. Even when compared to non-iterative classical filtering methods, such as the Wiener filter, which exhibits much lower latency compared to iterative methods, the competitive denoising performance of diffractive processors coupled with their efficiency in terms of speed, storage, and power consumption would be beneficial in many applications, particularly those demanding ultra-fast processing of scenes.

In this work, the diffractive image denoiser models were tested across various levels of different noise types. As shown in Figs. 2–5, the synthesized images at the output of the diffractive denoisers exhibit varying degrees of blur as a function of the input noise level or the output diffraction efficiency targeted during training. For example, Fig. 4 illustrates a trade-off between the denoising performance and image quality. A similar trade-off mechanism emerges between the image quality and the output diffraction efficiency of the denoiser models, as depicted in Fig. 5. This analysis reveals that a targeted increase in the output diffraction efficiency leads to a reduction in the sharpness of the denoised images for a given number of diffractive degrees of freedom that can be optimized. A better trade-off curve/relationship between the image filtering performance and the output diffraction efficiency can be achieved by increasing the number of trainable features and layers of a diffractive model.

In general, diffractive visual processors can perform any arbitrary complex-valued transformation between their input and output FoVs with negligible error if they have a sufficient number of trainable diffractive features, which scales with the multiplication of the number of useful pixels at the input and output FoVs^61–63^. For diffraction-limited operation, the lateral size of each diffractive feature should be ~λ/2, controlling all the propagating modes in air within the diffractive processor volume. However, fabricating a multi-layer diffractive visual processor with phase elements densely packed with a lateral feature size of ~λ/2 is challenging, especially for visible and IR wavelengths, due to the tight alignment and fabrication requirements. To mitigate these challenges and develop 3D fabrication processes optimized for diffractive network models, there have been various efforts to fabricate 3D diffractive networks operating in the visible and IR wavelengths^64–66^. To bring the presented framework to shorter wavelengths in, e.g., the visible band, different methods of 3D nano-fabrication, such as two-photon polymerization and optical lithography, can be used to manufacture and align the resulting diffractive layers of a diffractive image denoiser. In addition to these, vaccination strategies^49,50^ have been introduced to mitigate the impact of fabrication errors and physical misalignments by intentionally (and randomly) introducing such variations to the layers of a diffractive model during the training process to have more robust diffractive systems that can better withstand physical imperfections. One disadvantage of such vaccination efforts is that the independent degrees of freedom within the diffractive processor are reduced since the vaccination process effectively increases the feature size at the diffractive layer; this, however, can be mitigated by using wider and deeper architectures involving, e.g., a larger number of diffractive layers that are each wider.

Another limitation of the presented approach is that we only considered monochromatic illumination that is spatially coherent. While this assumption can be justified for certain applications that utilize, e.g., holographic image projection/display set-ups, it is also possible to extend the design of all-optical image denoisers to operate under spatially and temporally incoherent light. Diffractive optical networks, in general, form diffraction-limited universal linear transformers between an input and output FoV, and can be trained using deep learning to operate at various illumination wavelengths^59,67–71^, also covering spatially incoherent illumination^54^. Therefore, all-optical image denoisers and the underlying design framework can be extended to filter/denoise color images (e.g., RGB) or even spatially and temporally incoherent hyperspectral image signals.

In summary, we presented power-efficient and ultra-high speed all-optical image denoisers that filter out input image noise in the analog domain without consuming any power except for the illumination source. The success of all-optical image denoisers can inspire the creation of all-optical visual processors crafted to solve various other inverse problems in imaging and sensing.

## Materials and methods

### Diffractive image denoiser design

An all-optical image denoiser contains a series of diffractive surfaces $l=\mathrm{0,1},\ldots ,L-1$, each of which is located at a different axial position *z*_*l*_. The field transmittance of each diffractive surface ${T}_{l}\left(x,y\right)$ that is used to modulate the coherent wavefield ${U}_{l}\left(x,y\right)$ is stochastically optimized using deep learning^72^. The modulated coherent wavefield ${{U\text{'}}}_{l}\left(x,y\right)={U}_{l}\left(x,y\right){T}_{l}\left(x,y\right)$ is propagated to the axial position of next diffractive layer ${z}_{l+1}$ using the angular spectrum method, based on the Rayleigh-Sommerfeld diffraction integral that represents a 2D linear convolution of the propagation kernel $w(x,y,z)$ and the modulated wavefield ${{U\text{'}}}_{l}\left(x,y\right)$:

$${U}_{l+1}\left(x,y\right)={U^{\prime} }_{l}\left(x,y\right)* w(x,y,{{\rm{z}}}_{l+1}-{z}_{l})$$

$$w(x,y,z)=\frac{z}{{r}^{2}}\left(\frac{1}{2\pi r}+\frac{1}{{\rm{j}}\lambda }\right)\exp \left({\rm{j}}\frac{2\pi r}{\lambda }\right)$$

1 $$r=\sqrt{{x}^{2}+{y}^{2}+{z}^{2}}$$

where ${U}_{l+1}\left(x,y\right)$ denotes the coherent wavefield at the axial position ${z}_{l+1}$. The field transmittance function of each surface ${T}_{l}\left(x,y\right)$ is defined as:

2 $${T}_{l}\left(x,y\right)=\exp \left(j\frac{2\pi }{\lambda }\left(\tau \left(\lambda \right)-{n}_{a}\right){H}_{l}\left(x,y\right)\right)$$

where $\tau \left(\lambda \right)=n\left(\lambda \right)+{\rm{j}}\kappa \left(\lambda \right)$ is the complex refractive index of the optical material, ${n}_{a}=1$ refers to the refractive index of the medium (air in our case) surrounding the layers, and ${H}_{l}\left(x,y\right)$ represents the thickness profile of the corresponding diffractive surface, which is defined as

3 $${H}_{l}\left(x,y\right)={O}_{l}\left(x,y\right){(h}_{m}-{h}_{b})+{h}_{b}$$

where ${O}_{l}\left(x,y\right)$ is an auxiliary variable array used to compute the thickness value for each $\left(x,y\right)$ point between $[{h}_{b},{h}_{m}]$. ${O}_{l}\left(x,y\right)$ and consequently the thickness profile ${H}_{l}\left(x,y\right)$ for each diffractive layer *l* are jointly optimized using deep learning to obtain field transmittance function ${T}_{l}\left(x,y\right)$ for each surface^72–74^.

### Vaccination of the diffractive image denoisers

To mitigate the impact of potential misalignments in the physical implementation of a diffractive processor, error sources were integrated into the forward model during the training of the diffractive design that was experimentally demonstrated. These error sources are modeled by 3D displacement vectors, ${D}^{l}=({D}_{x},{D}_{y},{D}_{z})$ corresponding to the difference in the position of diffractive layer *l*, from its ideal location, where ${D}_{x},{D}_{y},$ and *D*_*z*_ were defined as uniformly distributed random variables,

4 $$\begin{array}{*{20}{l}}{D}_{x} \sim U(-{\Delta }_{x},{\Delta }_{x})\\{D}_{y} \sim U(-{\Delta }_{y},{\Delta }_{y})\\{D}_{z} \sim U(-{\Delta }_{z},{\Delta }_{z})\end{array}$$

where ∆_*x*_, ∆_*y*_, and ∆_*z*_ represents the maximum displacements along the *x*-, *y*-, and *z*- axes, respectively. Thus, the position of the diffractive layer *l* at *i*^th^ iteration *L*^(*l*,*i*)^ is defined as

5 $${L}^{(l,i)}=\left({L}_{x}^{l},{L}_{y}^{l},{L}_{z}^{l},\right)+\left({D}_{x}^{(l,i)},{D}_{y}^{(l,i)},{D}_{z}^{(l,i)}\right)$$

### Training and testing datasets

In our numerical results, we used 72,000 randomly selected images from the quickdraw dataset^45^. These images (28 × 28 pixels) were augmented by random rotations (ϴ $\sim U\left(-15^\circ ,15^\circ \right)$) and padded to 32 × 32 pixels. Then, they were split into three sets of images including 60,000 training, 2000 validation, and 10,000 test images. The prepared dataset is called *tiny quickdraw dataset*. To analyze the external generalization of the trained models, we also tested the resulting diffractive designs with unseen images from datasets different from the *tiny quickdraw dataset* including 14,400 EMNIST handwritten letters test images (interpolated to 32 × 32 using bicubic kernel) and 10,000 Fashion MNIST test images (scaled by 0.8 and interpolated to 32 × 32)^47,48^.

For the experimentally demonstrated design, the EMNIST dataset was used, which was split into two sets containing 80,000 training and 8,800 validation images. These datasets, along with the EMNIST test dataset (14,400 images) were interpolated to 20 × 20 pixels and used for the optimization and evaluation of experimentally-tested diffractive image denoiser. Without loss of generality, the contrasts of the images were inverted to facilitate the 3D fabrication of noisy objects in our experiments.

### Implementation details of all-optical denoisers for the numerical results

The smallest feature size of a transmissive diffractive layer and the sampling period of the propagation model were chosen as 0.5*λ*. Input and output FoVs of the diffractive image denoisers were 16*λ* × 16*λ* (32 × 32 in pixels). In addition, the size of each diffractive layer was chosen as 64*λ* × 64*λ* (128 × 128 in pixels). The window size of the propagation model was defined as 256 × 256 in pixels, and the matrices representing the FoVs and the diffractive layers were padded with zeros to avoid aliasing. In the numerical simulations, the material absorption was assumed to be zero (*κ*(*λ*) = 0), which is a valid assumption considering the overall thickness of our diffractive processor, axially spanning <250 × λ. The axial distance between two consecutive planes was chosen as 40*λ*. The phase coefficient function of each layer ${\theta }_{l}\left(x,y\right)=\frac{2\pi }{\lambda }\left(n\left(\lambda \right)-{n}_{a}\right){H}_{l}\left(x,y\right)$ and consequently the field transmittance function ${T}_{l}\left(x,y\right)$ were stochastically optimized using deep learning. ${\theta }_{l}\left(x,y\right)$ were initialized as 0 for each layer.

### Implementation details of the experimental results

A monochromatic THz illumination source (*λ* = ~0.75 mm) was used in the experiments. Input/output FoVs were determined to be 40*λ* × 40*λ* (3 cm × 3 cm) and the size of each diffractive layer was selected as 66.67*λ* × 66.67*λ* (5 cm × 5 cm). The diffractive feature width of the layers and the sampling period of the propagation model were chosen as ~0.667*λ*. The pixel size at the measurement plane was ~1.33*λ*, which is equivalent to the noise feature size at the input FoV. To accurately fabricate the transmissive diffractive layers and the noisy/clean input objects, the complex refractive index of the 3D-printing material *τ*(*λ*) was measured as ~1.6518 + j0.0612. During the training of the experimentally-tested diffractive image denoiser, the thickness profile of each trainable layer ${H}_{l}\left(x,y\right)$ was optimized in the range [0.5 mm,~1.65 mm] that corresponds to $[-\pi ,\pi )$ for phase modulation. For experimental testing, a 3-layer all-optical image denoiser was trained, fabricated, and tested. The axial distance between the input plane and the first diffractive layer was set to ~13.34*λ*. The other axial distances between successive layers were chosen as ~66.67*λ*. To have a misalignment-resilient design, the positions of the layers and the object were randomly shifted during training following the vaccination strategy outlined in Eq. 5. The maximum axial and lateral misalignments ∆_*x*_, ∆_*y*_, and ∆_*z*_ were chosen as ~0.26*λ*, ~0.26*λ*, and ~0.5*λ*, respectively. The thickness profiles of the trained diffractive surfaces and noisy/clean input objects were converted into STL files using MATLAB and they were fabricated by using a 3D printer (Objet30 Pro, Stratasys Ltd.).

The schematic diagram of the experimental setup is shown in Fig. 6d. The incident wave was generated using a modular amplifier (Virginia Diode Inc. WR9.0 M SGX)/multiplier chain (Virginia Diode Inc. WR4.3 × 2 WR2.2 × 2) (AMC) with a compatible diagonal horn antenna (Virginia Diode Inc. WR2.2). An RF input signal of 10 dBm at 11.1111 GHz (*f*_*RF1*_) generated by a synthesizer (HP 8340B) was used as the input and multiplied 36 times to produce continuous-wave (CW) radiation at 0.4 THz. The AMC was modulated with a 1 kHz square wave for lock-in detection. The axial distance between the exit aperture of the horn antenna and the object plane of the 3D-printed diffractive image denoiser was ~75 cm and the aperture of the horn antenna is measured to be ~4 mm × 4 mm. The output FoV of the diffractive denoiser was scanned using a 0.25 × 0.5 mm detector with a step size of 0.75 mm. To enhance the Signal-to-Noise Ratio (SNR) and better align with the output pixel size of our design, which was ~1.33λ (1 mm), a 3 × 3 bilinear upsampling and 4 × 4-pixel binning were used. The signals were detected by a Mixer (Virginia Diode Inc. WRI 2.2) equipped with a pinhole (0.25 × 0.5 mm) placed on an XY positioning stage composed of vertically combined two linear motorized stages (Thorlabs NRT100). A 10 dBm RF signal at 11.0833 GHz (*f*_*RF2*_) was sent to the detector as a local oscillator to down-convert the signal to 1 GHz for further measurement. The down-converted signal was amplified by a low-noise amplifier (Mini-Circuits ZRL-1150-LN+) and filtered using a 1 GHz (+/−10 MHz) bandpass filter (KL Electronics 3C40-1000/T10-O/O). The signal was initially measured by a low-noise power detector (Mini-Circuits ZX47-60) and read by a lock-in amplifier (Stanford Research SR830) with the 1 kHz square wave serving as the reference signal. The raw data were subsequently calibrated into a linear scale.

### Image noise models

In this work, two main noise models, namely salt and pepper noise^2^ and low sampling noise of Monte Carlo renderings^8,9^, were used to demonstrate the all-optical denoising performance of diffractive image denoisers for phase-encoded and intensity-encoded input objects. The former noise model represents a common noise type caused by abrupt and pronounced fluctuations within pixel values^2,75^. For both phase-encoded and intensity-encoded image denoising, the noise probability per image, ***P***_***tr***_ for training and ***P***_***te***_ for testing, determines the ratio of the pixels affected by salt or pepper noise relative to the overall pixel count within the image. Either salt or pepper noise is selected with a probability of 0.5. The pixel value of the salt is 1 for intensity images and *π* for phase images. The pixel value of the pepper is 0 for both intensity and phase images. We also used a modified version of the salt and pepper noise with 4 possible pixel values including 0, 0.33*π*, 0.66*π*, and *π*; see Supplementary Fig. S3b. Each one of these 4 pixel values is selected with a probability of 0.25. In the experimentally-tested design, only salt noise with a probability of 1 is used to simplify the fabrication of the noisy input objects in our experiments.

The second noise model involves image artifacts from Monte Carlo low-sample renderings, which poses an important problem for interactive ray tracing in real-world applications^9^. Unlike the salt and pepper noise, the noise model for ray tracing with low samples per pixel depends on pixel values and their positions in an image. To apply this noise model to input images, first, additive uniform noise $n(x,y) \sim U(\mathrm{0,0.5})$ is applied to every nonzero pixel. Following that, some pixels are impacted and set to 0 based on the noise probability per pixel ${{\boldsymbol{P}}}_{{\boldsymbol{\gamma }}}(x,y)$ that depends on the pixel location (*x*, *y*) and ***γ***. ${{\boldsymbol{P}}}_{{\boldsymbol{\gamma }}}(x,y)$ can be written as follows:

$${{\boldsymbol{P}}}_{{\boldsymbol{\gamma }}}\left(x,y\right)=\left\{\begin{array}{c}1-\frac{{r}_{\max }}{{\boldsymbol{\gamma }}r\left(x,y\right)},{\boldsymbol{\gamma }}r\left(x,y\right)\ge {r}_{\max }\\ 0,\qquad{otherwise}\end{array}\right.$$

$${r}_{\max }=\sqrt{{{x}_{\max }}^{2}+{{y}_{\max }}^{2}}$$

6 $$r(x,y)=\sqrt{{x}^{2}+{y}^{2}}$$

where *r*_max_ denotes the radius of a circle that encloses the image/object. *r*(*x*, *y*) refers to the distance between each pixel (*x*, *y*) and the center of the image. ***γ*** sets the noise rate of a poorly rendered image. ***γ***_***tr***_ and ***γ***_***te***_ are used to represent the noise rates for the training and testing phases, respectively.

### Training loss function and imaging performance comparison metrics

The loss function used for the training of our diffractive image denoiser models can be written as:

$${\mathcal{L}}=\frac{1}{N}\mathop{\sum }\limits_{i=1}^{N}\left|{y}_{i}-{\frac{{\sum }_{i=1}^{N}{y}_{i}}{{\sum }_{i=1}^{N}{\hat{y}}_{i}}{\hat{y}}_{i}}\right|+\beta {e}^{-\eta }$$

7 $$\eta =100\times \frac{{P}_{o}}{{P}_{i}}$$

where *y*_*i*_ and ${\hat{y}}_{i}$ represent each target image (ground truth) and the corresponding diffractive image denoiser output intensity, respectively. *N* denotes the number of pixels in each image. *P*_*i*_ and ${P}_{o}={\sum }_{i=1}^{N}{\hat{y}}_{i}$ are the optical power incident on the input FoV and the output FoV, respectively. *β* is used to adjust the average diffraction efficiencies of the all-optical image denoisers, which was set to be 0.05 for the experimentally-tested diffractive model and 0 for the numerically-tested designs, except for those presented in Fig. 5, which used different *β* values during their training to analyze the trade-off between the image denoising performance and the output diffraction efficiency.

To increase the generalization of the trained diffractive image denoisers, several data augmentation strategies including random image rotations (0, 90, 180, and 270 degrees), random flipping, and random contrast adjustments were incorporated into the training process. Python (v3.8.13) programming language and TensorFlow (v2.5.0, Google LLC) were used for the design of the all-optical image denoisers. The models were trained using a GeForce RTX 3090 GPU (Nvidia Corp.) and an AMD Ryzen Threadripper 3960X CPU (Advanced Micro Devices Inc.) with 264 GB of RAM.

To ensure a fine balance between the exploration and exploitation phases during the optimization process, we used cosine decay with warm-up to schedule our learning rate^76^. This scheduler initially increases the learning rate linearly from a predefined minimum value of *η*_min_ to a maximum value of *η*_max_ over a specified number of warm-up epochs ${T}_{{warm}-{up}}$ as follows:

8 $${\eta }_{t}={\eta }_{\min }+\frac{\left({\eta }_{\max }-{\eta }_{\min }\right)}{{T}_{{warm}-{up}}}\times t$$

where η_*t*_ represents the learning rate at epoch *t*. Then, for the remaining epochs (${t > T}_{{warm}-{up}}$), the learning rate starts to decrease with cosine decay as

9 $${\eta }_{t}={\eta }_{\min }+\frac{1}{2}\left({\eta }_{\max }-{\eta }_{\min }\right)\left(1+\cos \left(\frac{\pi \times \left(t-{T}_{{warm}-{up}}\right)}{T-{T}_{{warm}-{up}}}\right)\right)$$

where *T* denotes the total number of epochs. *η*_max_ and *η*_min_ were set to be 0.002 and 0. For the numerically-tested designs, *T* and ${T}_{{warm}-{up}}$ were 500 and 62 epochs, respectively. For experimentally-demonstrated design, *T* and ${T}_{{warm}-{up}}$ were 150 and 22 epochs, respectively. All models were trained using the Adam optimizer with a batch size of 200. Each of the numerically-tested designs utilized ~12 h of training, spanning 500 epochs.

To quantify our denoised imaging results, PSNR and SSIM metrics were used to compare the all-optical image denoising performance of different diffractive designs^46^. For the SSIM computation, the standard TensorFlow implementation with a maximum value of 1 was used, and for the PSNR computation we used:

10 $${PSNR}=10{\log }_{10}\left(\frac{1}{\frac{1}{N}{\sum }_{i=1}^{N}{\left|{y}_{i}-{\frac{{\sum }_{i=1}^{N}{y}_{i}}{{\sum }_{i=1}^{N}{\hat{y}}_{i}}{\hat{y}}_{i}}\right|}^{2}}\right)$$

### Supplementary information

Supplementary Information

## References

[1] Bertero, M., Boccacci, P. & De Mol, C. *Introduction to Inverse Problems in Imaging* (CRC Press, 2021).

[2] Gonzalez, R. C. & Woods, R. E. *Digital Image Processing* (Pearson Education, 2009).

[3] Haines, E. & Akenine-Möller, T. *Ray Tracing Gems: High-Quality and Real-Time Rendering with DXR and Other APIs* (Apress, 2019), 10.1007/978-1-4842-4427-2

[4] Blinder D (2022). The state-of-the-art in computer generated holography for 3D display. Light.: Adv. Manuf..

[5] Bai B (2022). To image, or not to image: class-specific diffractive cameras with all-optical erasure of undesired objects. eLight.

[6] Lundberg, K. H. Noise Sources in Bulk CMOS. https://www.mit.edu/people/klund/papers/UNP_noise.pdf (2002).

[7] Noise, dynamic range and bit depth in digital SLRs. https://homes.psd.uchicago.edu/~ejmartin/pix/20d/tests/noise/ (2023).

[8] Chaitanya CRA (2017). Interactive reconstruction of Monte Carlo image sequences using a recurrent denoising autoencoder. ACM Trans. Graph..

[9] Işık M (2021). Interactive Monte Carlo denoising using affinity of neural features. ACM Trans. Graph..

[10] Introducing the NVIDIA RTX Ray Tracing Platform. NVIDIA developer. at https://developer.nvidia.com/rtx/ray-tracing (2018).

[11] Elad M, Kawar B, Vaksman G (2023). Image denoising: The deep learning revolution and beyond - a survey paper. SIAM J. Imaging Sci..

[12] Buades A, Coll B, Morel JM (2005). A review of image denoising algorithms, with a new one. Multiscale Model. Simul..

[13] Milanfar P (2013). A tour of modern image filtering: new insights and methods, both practical and theoretical. IEEE Signal Process. Mag..

[14] Lindenbaum M, Fischer M, Bruckstein A (1994). On Gabor’s contribution to image enhancement. Pattern Recognit..

[15] Perona P, Malik J (1990). Scale-space and edge detection using anisotropic diffusion. IEEE Trans. Pattern Anal. Mach. Intell..

[16] Rudin LI, Osher S, Fatemi E (1992). Nonlinear total variation based noise removal algorithms. Phys. D: Nonlinear Phenom..

[17] Donoho DL (1995). De-noising by soft-thresholding. IEEE Trans. Inf. Theory.

[18] Tomasi, C. & Manduchi, R. Bilateral filtering for gray and color images. In *Sixth International Conference on Computer Vision (IEEE Cat. No.98CH36271)*. 839–846 (IEEE, 1998), 10.1109/I1998.710815.

[19] Buades, A., Coll, B. & Morel, J. M. A non-local algorithm for image denoising. In *2005 IEEE Computer Society Conference on Computer Vision and Pattern Recognition (CVPR’05)*. 60–65 (IEEE, 2005), 10.1109/C2005.38.

[20] Dabov K (2007). Image denoising by sparse 3-D transform-domain collaborative filtering. IEEE Trans. Image Process..

[21] Lucas A (2018). Using deep neural networks for inverse problems in imaging: beyond analytical methods. IEEE Signal Process. Mag..

[22] McCann MT, Jin KH, Unser M (2017). Convolutional neural networks for inverse problems in imaging: a review. IEEE Signal Process. Mag..

[23] Zhang K (2017). Beyond a Gaussian denoiser: residual learning of deep CNN for image denoising. IEEE Trans. Image Process..

[24] Burger, H. C., Schuler, C. J. & Harmeling, S. Image denoising: Can plain neural networks compete with BM3D? In *2012 IEEE Conference on Computer Vision and Pattern Recognition*. 2392–2399 (IEEE, 2012), 10.1109/CVPR.2012.6247952.

[25] Plötz, T. & Roth, S. Neural nearest neighbors networks. In *Proceedings of the 32nd International Conference on Neural Information Processing Systems* (Curran Associates Inc., 2018).

[26] Valsesia D, Fracastoro G, Magli E (2020). Deep graph-convolutional image denoising. IEEE Trans. Image Process..

[27] Liang, J. Y. et al. SwinIR: Image restoration using swin transformer. In *Proceedings of the 2021 IEEE/CVF International Conference on Computer Vision Workshops*. 1833–1844 (IEEE, 2021).

[28] Zhang K, Zuo WM, Zhang L (2018). FFDNet: Toward a fast and flexible solution for CNN-based image denoising. IEEE Trans. Image Process..

[29] Tu, Z. et al. MAXIM: Multi-axis MLP for image processing. In *Proceedings of the 2022 IEEE/CVF Conference on Computer Vision and Pattern Recognition*. 5759–5770 (IEEE, 2022).

[30] Wang, Z. D. et al. Uformer: a general U-shaped transformer for image restoration. In *Proceedings of the IEEE/CVF Conference on Computer Vision and Pattern Recognition*. 17662–17672 (IEEE, 2022).

[31] Li YH (2023). Quantitative phase imaging (QPI) through random diffusers using a diffractive optical network. Light.: Adv. Manuf..

[32] Situ GH (2022). Deep holography. Light.: Adv. Manuf..

[33] Lin H, Cheng JX (2023). Computational coherent Raman scattering imaging: breaking physical barriers by fusion of advanced instrumentation and data science. eLight.

[34] Koskela M (2019). Blockwise multi-order feature regression for real-time path-tracing reconstruction. ACM Trans. Graph..

[35] Hasselgren J (2020). Neural temporal adaptive sampling and denoising. Comput. Graph. Forum.

[36] Meng, X. X. et al. Real-time Monte Carlo Denoising with the neural bilateral grid. In *31st Eurographics Symposium on Rendering* (Eurographics Association, 2020).

[37] Mengu D (2020). Analysis of diffractive optical neural networks and their integration with electronic neural networks. IEEE J. Sel. Top. Quantum Electron..

[38] Justus, D. et al. Predicting the computational cost of deep learning models. In 2018 *IEEE International Conference on Big Data (Big Data)*. 3873–3882, (IEEE, 2018), 10.1109/BigData.2018.8622396.

[39] Luo Y (2022). Computational imaging without a computer: seeing through random diffusers at the speed of light. eLight.

[40] Wang TY (2023). Image sensing with multilayer nonlinear optical neural networks. Nat. Photonics.

[41] Wetzstein G (2020). Inference in artificial intelligence with deep optics and photonics. Nature.

[42] Goi E, Schoenhardt S, Gu M (2022). Direct retrieval of Zernike-based pupil functions using integrated diffractive deep neural networks. Nat. Commun..

[43] Chen YT (2023). Photonic unsupervised learning variational autoencoder for high-throughput and low-latency image transmission. Sci. Adv..

[44] Mengu D, Ozcan A (2022). All-optical phase recovery: diffractive computing for quantitative phase imaging. Adv. Optical Mater..

[45] Ha, D. & Eck, D. A neural representation of sketch drawings. In *6th International Conference on Learning Representations*. (OpenReview.net, 2018).

[46] Wang Z (2004). Image quality assessment: from error visibility to structural similarity. IEEE Trans. Image Process..

[47] Xiao, H., Rasul, K. & Vollgraf, R. Fashion-MNIST: a novel image dataset for benchmarking machine learning algorithms. Preprint at https://arxiv.org/abs/1708.07747.

[48] Cohen, G. et al. EMNIST: Extending MNIST to handwritten letters. In 2017 *International Joint Conference on Neural Networks (IJCNN)*. 2921–2926 (IEEE, 2017). 10.1109/IJCNN.2017.7966217.

[49] Mengu D (2020). Misalignment resilient diffractive optical networks. Nanophotonics.

[50] Mengu D, Rivenson Y, Ozcan A (2021). Scale-, shift-, and rotation-invariant diffractive optical networks. ACS Photonics.

[51] Wang J (2022). Speckle suppression using F-D^2^NN in holographic display. Displays.

[52] Deng YB, Chu DP (2017). Coherence properties of different light sources and their effect on the image sharpness and speckle of holographic displays. Sci. Rep..

[53] Li JX (2023). Unidirectional imaging using deep learning–designed materials. Sci. Adv..

[54] Rahman MSS (2023). Universal linear intensity transformations using spatially incoherent diffractive processors. Light Sci. Appl..

[55] Gharbi M (2017). Deep bilateral learning for real-time image enhancement. ACM Trans. Graph..

[56] Tang, Y. H. et al. GhostNetV2: Enhance cheap operation with long-range attention. In *Proceedings of the 36th International Conference on Neural Information Processing Systems* (neurips.cc, 2022).

[57] Desislavov R, Martínez-Plumed F, Hernández-Orallo J (2023). Trends in AI inference energy consumption: Beyond the performance-vs-parameter laws of deep learning. Sustain. Comput. Inform. Syst..

[58] Rahman MSS (2023). Learning diffractive optical communication around arbitrary opaque occlusions. Nat. Commun..

[59] Işıl Ç (2022). Super-resolution image display using diffractive decoders. Sci. Adv..

[60] Li YH (2023). Optical information transfer through random unknown diffusers using electronic encoding and diffractive decoding. Adv. Photonics.

[61] Kulce O (2021). All-optical information-processing capacity of diffractive surfaces. Light Sci. Appl..

[62] Kulce O (2021). All-optical synthesis of an arbitrary linear transformation using diffractive surfaces. Light Sci. Appl..

[63] Li JX (2022). Polarization multiplexed diffractive computing: all-optical implementation of a group of linear transformations through a polarization-encoded diffractive network. Light Sci. Appl..

[64] Goi E (2021). Nanoprinted high-neuron-density optical linear perceptrons performing near-infrared inference on a CMOS chip. Light Sci. Appl..

[65] Chen H (2021). Diffractive deep neural networks at visible wavelengths. Engineering.

[66] Bai BJ (2023). Data-class-specific all-optical transformations and encryption. Adv. Mater..

[67] Luo Y (2019). Design of task-specific optical systems using broadband diffractive neural networks. Light Sci. Appl..

[68] Li JX (2021). Spectrally encoded single-pixel machine vision using diffractive networks. Sci. Adv..

[69] Mengu D (2023). Snapshot multispectral imaging using a diffractive optical network. Light Sci. Appl..

[70] Shen CY (2023). Multispectral quantitative phase imaging using a diffractive optical network. Adv. Intell. Syst..

[71] Li JX (2023). Massively parallel universal linear transformations using a wavelength-multiplexed diffractive optical network. Adv. Photonics.

[72] Lin X (2018). All-optical machine learning using diffractive deep neural networks. Science.

[73] LeCun Y, Bengio Y, Hinton G (2015). Deep learning. Nature.

[74] Rumelhart DE, Hinton GE, Williams RJ (1986). Learning representations by back-propagating errors. Nature.

[75] Quan, Y. H. et al. Self2Self with dropout: learning self-supervised denoising from single image. In *Proceedings of the IEEE/CVF Conference on Computer Vision and Pattern Recognition*. 1887–1895 (IEEE, 2020).

[76] Loshchilov, I. & Hutter, F. SGDR: stochastic gradient descent with warm restarts. In *Presented at the 5th International Conference on Learning Representations* (OpenReview.net, 2017).

