# Supplementary material for: All-optical image denoising using a diffractive visual processor
Source: Light Sci Appl. 2024 Feb 4;13:43. doi: 10.1038/s41377-024-01385-6 (PMC10838318; doi:10.1038/s41377-024-01385-6)
Supplement: Supplementary file 1 — Supplementary Information [file 41377_2024_1385_MOESM1_ESM.pdf]

Supplementary Information for

All-optical image denoising using a diffractive visual processor

Çağatay Işıl[1,2,3], Tianyi Gan[1,3], F. Onuralp Ardic[1], Koray Mentesoglu[1], Jagrit Digani[1], Huseyin Karaca[1], Hanlong Chen[1,2,3], Jingxi Li[1,2,3], Deniz Mengu[1,2,3], Mona Jarrahi[1,3], Kaan Akşit[4], and Aydogan Ozcan[1,2,3,*]

[1]Electrical and Computer Engineering Department, University of California, Los Angeles, CA, 90095, USA

[2]Bioengineering Department, University of California, Los Angeles, CA, 90095, USA

[3]California NanoSystems Institute (CNSI), University of California, Los Angeles, CA, 90095, USA

[4]University College London, Department of Computer Science, London, United Kingdom

* ozcan@ucla.edu

This PDF file includes:

Supplementary Figs. S1-S4

Supplementary Figures

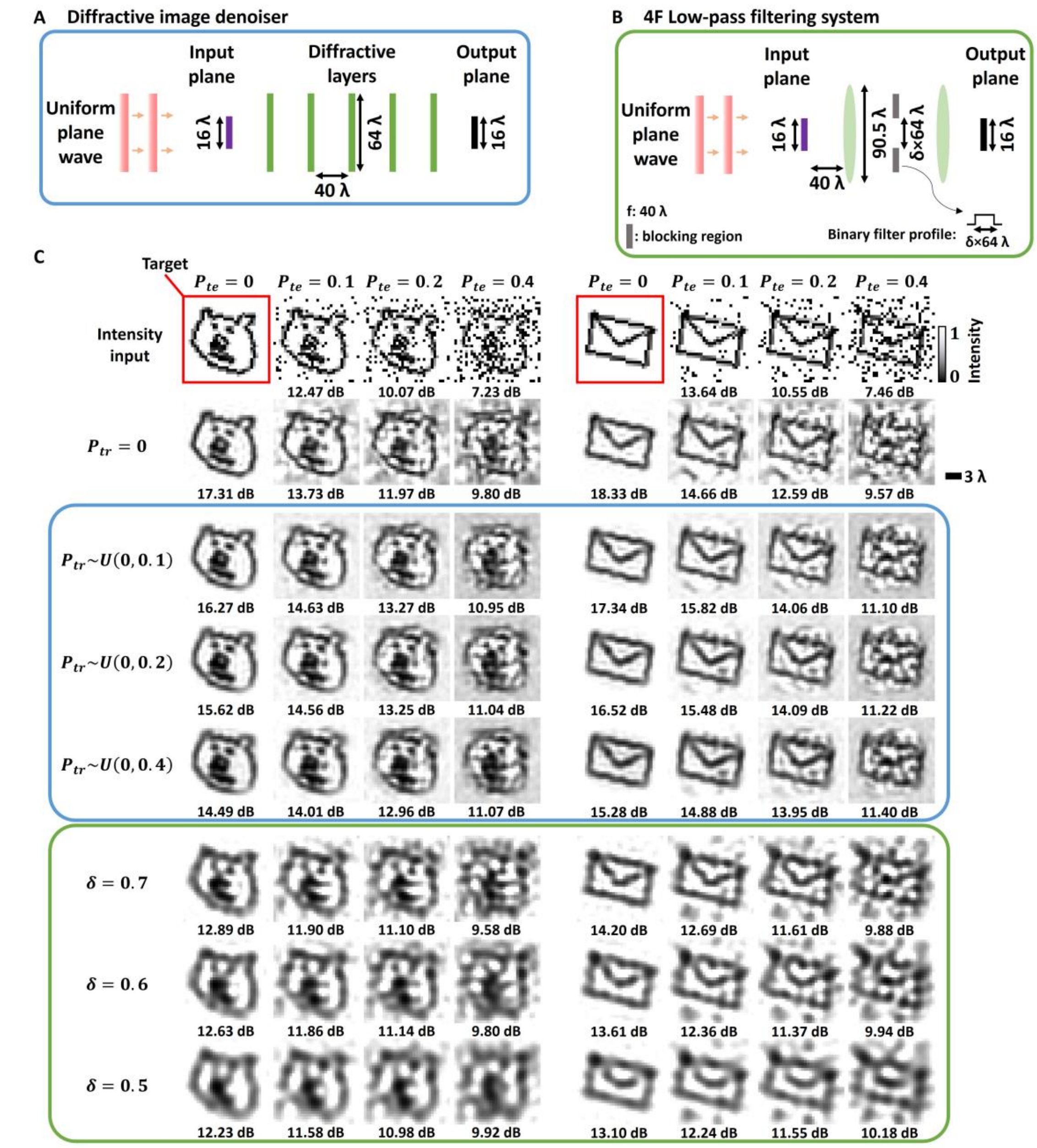

Figure S1. Simulation results of 5-layer all-optical diffractive image denoisers and a 4-f lens-based filtering system for filtering out salt & pepper noise. Same numerical aperture (NA) is used for the diffractive denoisers and the 4-f system. NA is calculated using the diagonal of the first diffractive layer and the diameter of the first lens for the diffractive denoisers and the 4-f system, respectively. **(A)** Optical layout of the diffractive image denoisers operating on intensity input images. **(B)** Optical layout of the 4-f lens-based filtering system operating on intensity input images. **(C)** All-optical image denoising results of different diffractive denoisers with intensity-encoded inputs, trained using $\boldsymbol{P_{tr}}$ drawn uniformly from different intervals, and 4-f lens-based filtering systems using different filtering ratios $\boldsymbol{\delta}$. The PSNR value for each case is shown beneath the respective image.

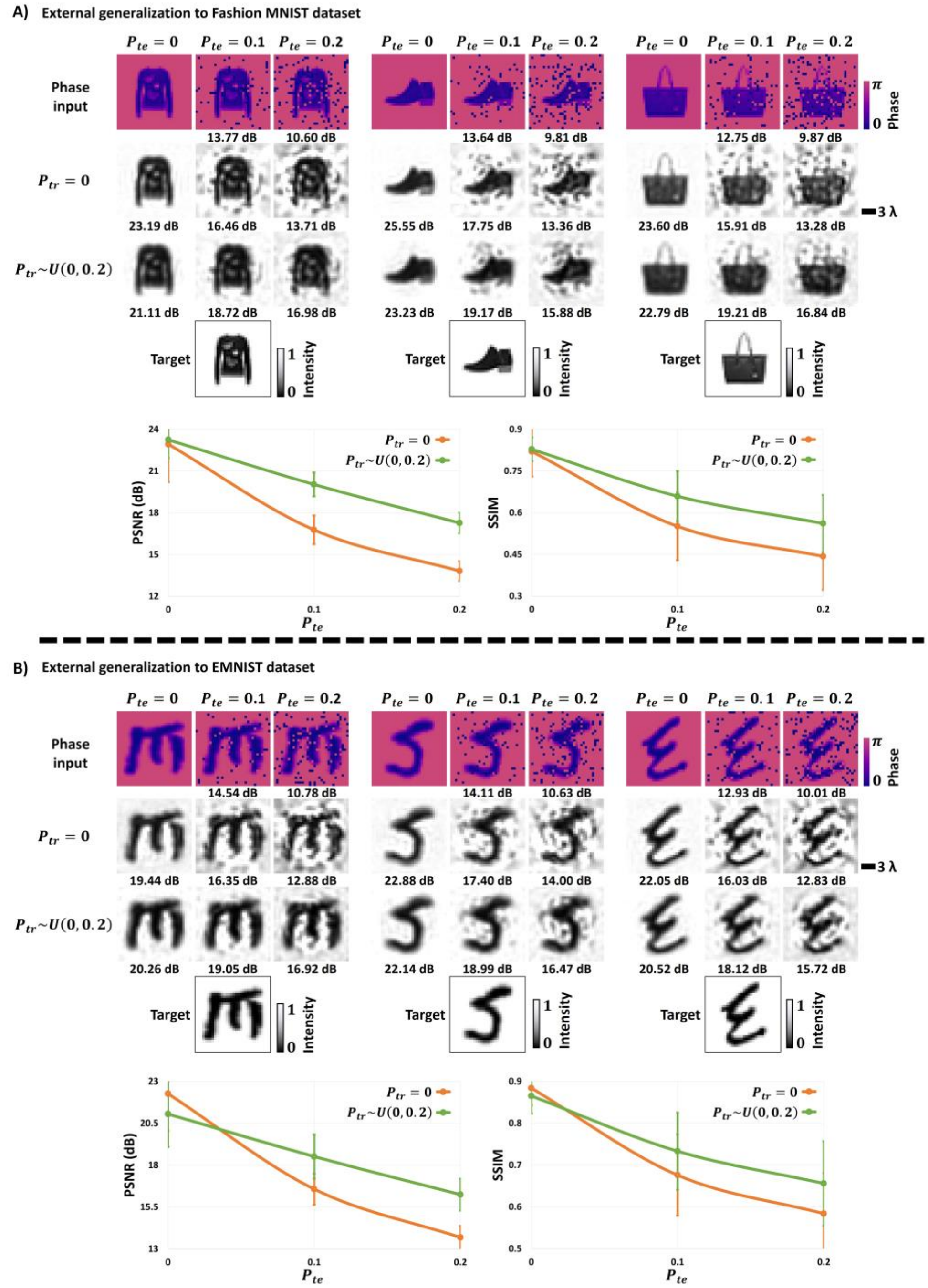

Figure S2. External generalization performance of 5-layer all-optical diffractive image denoisers for salt and pepper noise removal. The diffractive image denoiser using phase-encoded inputs is trained with the *tiny quickdraw* dataset [1]. **(A)** All-optical image denoising results on three images randomly selected from the Fashion MNIST test dataset [2] and the average PSNR and SSIM values on the same test dataset as a function of $\boldsymbol{P_{te}}$. The PSNR value for each case is shown beneath the corresponding image. **(B)** All-optical image denoising results on three images of the EMNIST test dataset [3] and the average PSNR and SSIM values on the same dataset as a function of $\boldsymbol{P_{te}}$.

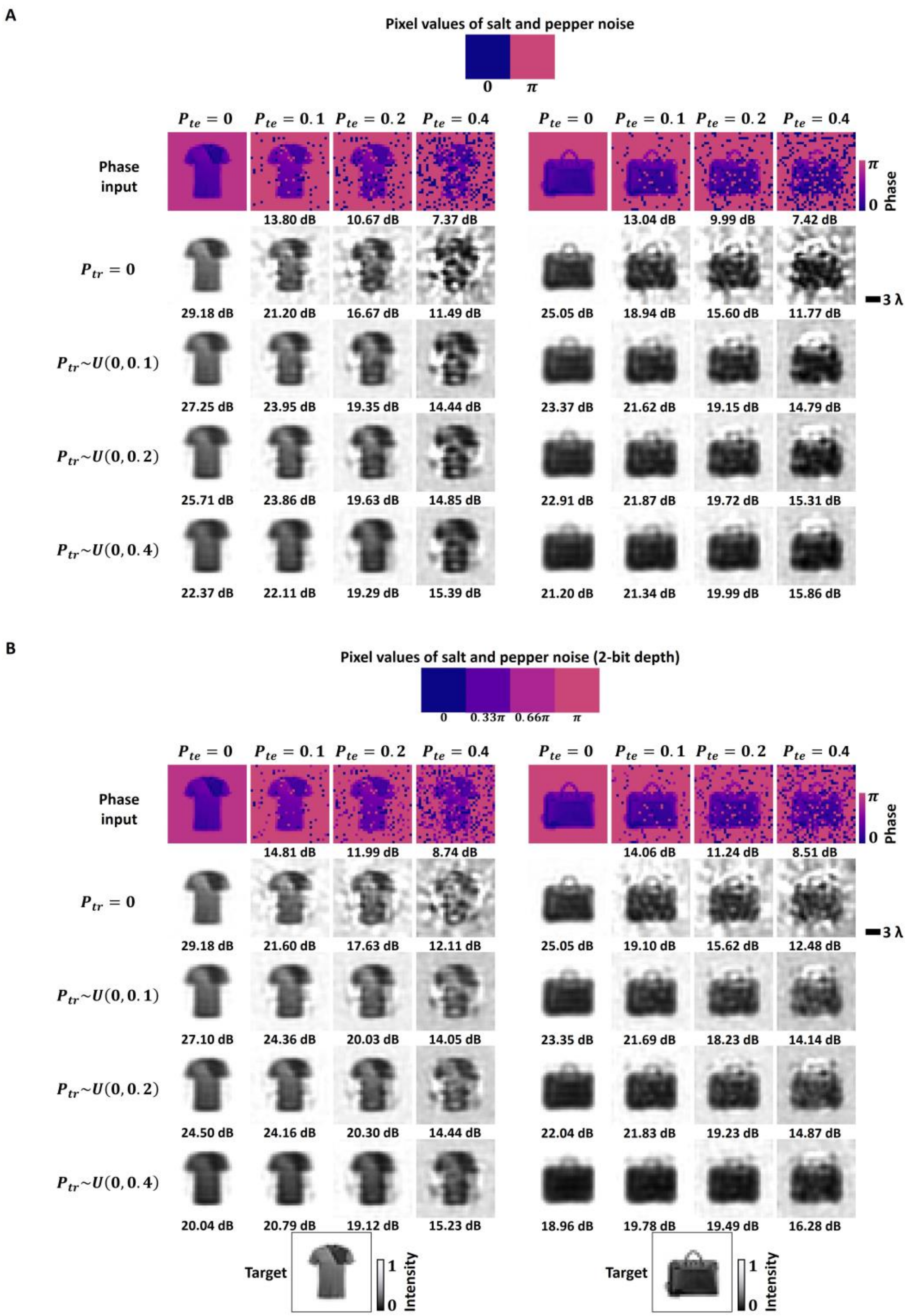

Figure S3. Simulation results of 5-layer all-optical diffractive image denoisers for filtering out salt & pepper noise and its 2-bit version (covering 4 levels). Diffractive image denoisers using phase-encoded inputs are trained using the Fashion MNIST image dataset. **(A)** All-optical image denoising results of different diffractive image denoisers, which are trained using P_{tr} drawn uniformly from different intervals for salt & pepper noise. **(B)** All-optical image denoising results of different diffractive denoisers, trained using P_{tr} drawn uniformly from different intervals for the 2-bit version of the salt & pepper noise. The PSNR value for each case is shown beneath the corresponding image.

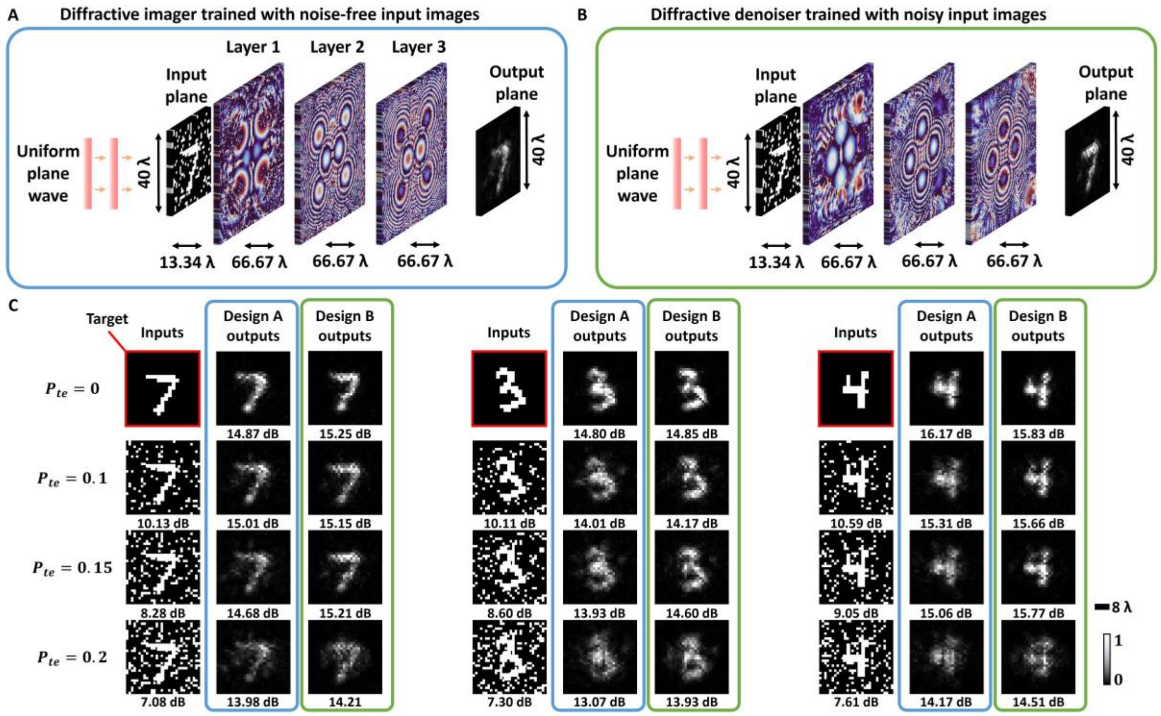

Figure S4. Simulation results of the diffractive imager trained with noise-free images and the diffractive image denoiser trained with noisy input images. During the training of these models composed of 3 transmissive diffractive layers, we used the same physical configuration and error sources for the misalignment between the layers. **(A)** Layout of the diffractive imager. **(B)** Layout of the diffractive image denoiser. **(C)** Image denoising performance of the designed diffractive denoiser and diffractive imager under different levels of salt-only noise ($\boldsymbol{P_{te}}$). The PSNR value for each case is shown beneath the corresponding image.

References

1. Ha, D. & Eck, D. A neural representation of sketch drawings. 6th International Conference on Learning Representations. Vancouver, BC, Canada: OpenReview.net, 2018.
2. Xiao, H., Rasul, K. & Vollgraf, R. Fashion-MNIST: a novel image dataset for benchmarking machine learning algorithms. Print at https://arxiv.org/abs/1708.07747.
3. Cohen, G. et al. EMNIST: Extending MNIST to handwritten letters. 2017 International Joint Conference on Neural Networks (IJCNN). Anchorage, AK, USA: IEEE, 2017, 2921-2926. doi: 10.1109/IJCNN.2017.7966217.
